# Medication Appropriateness in Prehospital Care

**DOI:** 10.1155/2019/6947698

**Published:** 2019-09-02

**Authors:** Nikolai Ramadanov, Roman Klein, Abner Daniel Aguilar Valdez, Wilhelm Behringer

**Affiliations:** ^1^Center for Emergency Medicine, University Hospital Jena, Friedrich Schiller University, Am Klinikum 1, 07747 Jena, Germany; ^2^Orthopaedics, Trauma Surgery and Sports Traumatology, Marienhaus Hospital Hetzelstift, Stiftstr. 10, 67434 Neustadt, Germany; ^3^Center for Internal Medicine, Clinic for Endocrinology and Diabetology, Ernst von Bergmann Hospital Bad Belzig, Niemegker Str. 45, 14806 Bad Belzig, Germany

## Abstract

**Background:**

The aim of the present study was to determine the medication appropriateness (MA) in prehospital emergency physician deployments according to the hospital discharge diagnosis and to investigate the factors influencing the MA.

**Methods:**

The MA was determined by a systematic comparison of the administered medication in prehospital emergency physician deployments with the discharge diagnosis in a period of 24 months at the emergency medical services in Bad Belzig. Categorial variables for the specialty, medical educational status, and approval for emergency medicine of prehospital emergency physicians were examined univariate for relations with the MA, using the *χ*2 test with the significance level of *p*=0.05.

**Results:**

The MA was present in 69% (*n* = 488) cases. The MA was present in 64% of cases by specialists and in 71% by resident physicians (*p*=0.04). The specialty and the approval for emergency medicine of the prehospital emergency physician did not show significant results. MA was present in 46% (*n* = 100) of cases with incorrect diagnoses, and it was present in 79% (*n* = 388) of cases with correct diagnoses by the prehospital emergency physician (*p*=0.01). In cases of missing MA, 224 drugs and 23 different drugs were administered by the prehospital emergency physician.

**Conclusions:**

The MA in prehospital emergency physician deployments shows a necessity for improvement with 31% medication errors. Incorrect diagnoses by the prehospital emergency physician seem to lead to medication errors in prehospital emergency physician deployments. The necessary standards and guidelines for administration of drugs should be taken into account in educational courses. The wide-ranging emergency medical training and the rapid accumulation of operational experience seem to play a crucial role for correct administration of medication in the prehospital emergency physician deployments.

## 1. Introduction

Medication errors comprehend a wide range of situations leading to inappropriate medication use or patient harm. Mostly a medication error is an inadvertent misprescription or administration of a drug [1]. In case of medication errors, there is the possibility of avoidance [2, 3]. Medication errors are not uncommon in the clinic. For example, 985 cases transferred from a total of 58 intensive care units to normal wards, and it was found that medication errors occurred in 45.7% [4]. A meta-analysis of 63 studies from 1985 to 2007 showed that every second hospital admission was affected by medication errors with a median value of 7% of all in-hospital medication orders [5]. According to calculations of the German Institute for Drugs and Medical Products, avoidable medication errors in Germany lead to around 500,000 hospital admissions each year [6]. A study in an emergency department in Madrid with 1839 patients in 2016 found that ¼ of the medication errors were potentially severely harmful [7]. The following factors leading to medication errors could be identified: lack of knowledge about drugs and their administration (30%), lack of knowledge about the patient (29%), incorrect calculations (18%), and problems with the nomenclature (13%) [8].

There are numerous studies on medication errors after treatment by in-hospital physicians [9–12]. The present study is intended to supplement the current literature with an examination of medication appropriateness after treatment by prehospital emergency physicians in the emergency medical service (EMS). Since prehospital emergency physician deployments often have a life-saving character, a medication error can have fatal consequences.

The aim of the present study was to determine the MA in prehospital emergency physician deployments according to the hospital discharge diagnosis and to investigate the factors influencing the MA.

## 2. Methods

### 2.1. Data Collection

Data were collected from all prehospital emergency physician's patient care reports (DIVIDOK 4.2) of the EMS Center in Bad Belzig in the period from July 1, 2013, to June 30, 2014, and from January 1, 2015, to December 31, 2015. The corresponding discharge summaries were taken from the hospital information system SAP version 7400.1.0.1093 in the hospital Bad Belzig as well as from neighboring hospitals (Klinikum Ernst von Bergmann Potsdam, Asklepios Fachkinikum Brandenburg, Städtisches Klinikum Brandenburg, and Johanniter-Krankenhaus im Fläming Treuenbrietzen). Patients or family members provided written informed consent, and the Ethical Committee of the University of Jena approved the data collection.

### 2.2. Determination of Medication Appropriateness

First, all prehospital emergency physician deployments with multiple deployment-related hospital discharge diagnoses were excluded since otherwise the clear assignment of the prehospital medication to the corresponding diagnosis is impossible. Through a systematic comparison of administered prehospital medication to hospital discharge diagnosis, the MA was determined. The determination of the MA was carried out by the consensus of three experienced prehospital emergency physicians. There was an interrater reliability of 0.96 for MA. In divergent cases, the judgment of the third emergency physician was consulted. Missing MA was noted when the administration of an omitted drug was obligatory for the corresponding discharge diagnosis according to current guidelines or if the administration of an unnecessary drug was contraindicated for the corresponding diagnosis according to current guidelines.

### 2.3. Definition and Classification of Medication Errors

In our study, medication errors were defined as cases with missing MA. Since the present study investigates only the administered medication, recorded in the patient care reports, all of the medication errors reached the patient. The present study included “errors with an adverse reaction” and “errors without harm,” according to the “Good practice guide on recording, coding, reporting and assessment of medication errors” of the European Medicines Agency, classified in correlation of harm and preventability. Because of the design of the study, “intercepted errors” and “potential errors” were not investigated.

The types of medication errors were classified as follows:

1. Omission of obligatory medication
2. Administration of contraindicated medication

The correctness of the dosage was not considered.

### 2.4. Determination of Diagnostic Agreement

The determination of the DA was carried out by the consensus of two experienced prehospital emergency physicians, using the ICD 10 coding. There was an interrater reliability of 1.0 for DA. The confirmation of the deployment-related hospital discharge diagnosis in the prehospital emergency physician's patient care reports was without consideration of the number of suspected diagnoses by the prehospital emergency physician. Only deployment-related hospital discharge diagnoses were considered; i.e., complications that occurred during the hospital stay were ignored as diagnoses in the discharge summaries.

### 2.5. Exclusion of Cases

Of the 1760 prehospital emergency physician deployments during the investigation period, 708 cases were included in the study. Patient care reports were excluded from the study for the following reasons: ambulant treatment in the emergency department (*n* = 323), prehospital treatment and lack of admission to the ED (*n* = 251), lack of recorded emergency diagnosis (*n* = 122), and death of the patient during the deployment (*n* = 42) or incorrect/unreadable patient data (*n* = 35). All cases with more than one deployment-related discharge diagnosis were excluded (*n* = 279).

### 2.6. Statistics

Statistical calculations were performed using IBM SPSS Statistics 19 for Windows (IBM Germany GmbH, Ehningen). The significance level was 0.05. In the course of the evaluation, the categorial variables for the specialty, medical educational status, and approval for emergency medicine of prehospital emergency physicians were examined univariate for relations with the categorial variables MA. In addition, the independence between the MA and DA was examined. For this purpose, the *χ*^2^ test was calculated.

## 3. Results

### 3.1. Patients

Of the 708 patients included, 336 were male and 370 were female. The mean age of the patients was 68 (standard deviation ± 20) years (min <1, max 97 years). 394 (84%) of the included patients were fully oriented, 51 (11%) clouded, and 26 (5%) unconscious. 180 (31%) patients regularly took ≤4 drug as home medication. 398 (69%) patients regularly took more than 4 drugs.

### 3.2. Prehospital Emergency Physicians

Table 1 shows the distribution of the prehospital emergency physician deployments according to specialty, medical educational status, and approval for emergency medicine of prehospital emergency physicians.

### 3.3. Description of the Medication

Table 2 shows the 10 most commonly used drugs in the 220 cases of missing MA. In cases of missing MA, 224 drugs and 23 different drugs were administered by the prehospital emergency physician. In total, 1058 drugs and 37 different drugs were administered by the prehospital emergency physicians.  Table 3 shows the administered drugs with missing MA, summarized in medication groups by ATC codes.

### 3.4. Spectrum of Discharge Diagnoses

Due to the abovementioned exclusion criteria, the study did not include cases with more than one deployment-related discharge diagnosis.  Table 4 shows the 10 most frequent deployment-related discharge diagnoses.

### 3.5. Medication Appropriateness

MA was present in 68.9% (*n* = 488) of cases. 49.1% (*n* = 108) of cases with missing MA was due to omission of obligatory medication, and 50.9% (*n* = 112) of cases with missing MA was due to administration of contraindicated medication.

### 3.6. Influence of Prehospital Emergency Physician-Specific Factors on MA

**Figure 1:**
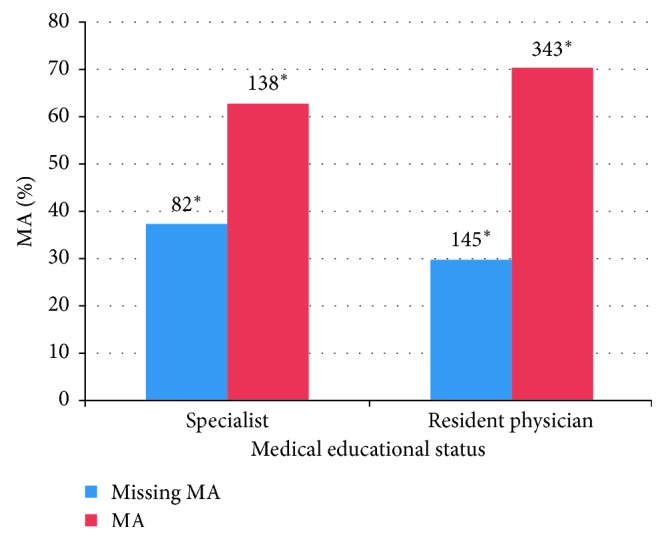
Representation of the MA taking into account the medical educational status of the prehospital emergency physician. *p*=0.04; ^*∗*^number of deployments.

Figure 1 shows the distribution of MA according to the medical educational status of prehospital emergency physicians. MA was present in 62.7% (*n* = 138) of cases by specialists and in 70.3% (*n* = 343) by resident physicians (*χ*^2^ = 4.0, DF = 1, *p*=0.04). The specialty (*χ*^2^ = 5.9, DF = 3, *p*=0.12) and the approval for emergency medicine of prehospital emergency physicians (*χ*^2^ = 1.9, DF = 1, *p*=0.16) had no statistically significant influence on MA.

### 3.7. Diagnostic Agreement and Correlation between MA and DA

The DA was present in 69.4% (*n* = 491) of cases. In the case of present DA, a MA was present in 79.0% (*n* = 388) of cases. In the case of lacking DA, a MA was present in 46.1% (*n* = 100) (*χ*^2^ = 76.2, DF = 1, *p*=0.01).

## 4. Discussion

The correctness of the administered medication could be estimated retrospectively in the present study through the calculation of the MA. Our investigation showed, based on 708 included cases, that the administered medication was incorrect in 31%. Several in-hospital studies show an even higher proportion of medication errors.

In a study with 200 adult patients about medication after admission in the emergency department, discrepancies were assessed through comparison of the home medication list with the physician's orders. 77.5% of patients had one or more medication discrepancies. The most common discrepancies were medication omission (35.49%) [9]. In another study with 200 cases, 47% was found to have at least one unintentional medication discrepancy. Among the unintentional discrepancies, 67% was associated with a potential harm to the patients. Increasing patients' age was significantly associated with higher number of discrepancies [10]. In a study with 100 pediatric patients, 13.0% contained at least one unintentional discrepancy. 84.6% of them were associated with mild potential harm to patients. 61.5% were omission of medications and 38.5% addition of unnecessary medication [11]. In another study with 832 patients, 11.7% experienced at least one medication discrepancy following admission to the hospital [12]. In a study of 352 paramedics surveyed, only 9.1% reported medication errors in prehospital deployments in the last 12 months. In 63% of those errors, the wrong dose and in 4% the wrong medication was administered [13].

However, as the present study examines the MA of emergency medication in prehospital care, the calculated proportion of 31% medication errors should still be viewed critically. This result raises the question of whether the necessary standards and guidelines for the administration of medication by the prehospital emergency physicians according to the diagnoses and clinical pictures of the patients were complied with.

As possible measures to avoid medication errors, some of which are also preclinical applicable, the following can be considered: strengthening the awareness of the patient and physician, improving communication between patient and physician, sufficient specialized information for the physician, involvement of pharmacists in the visit, consideration of the preexisting conditions and premedication of the patient, and promotion of a safety culture [2, 3]. In a prospective cohort comparison study, it was found that pharmacists led to fewer medication discrepancies than emergency physicians while admitting patients in the ED [14].

The medical educational status of prehospital emergency physicians showed a significant influence on the medication errors in prehospital emergency physician deployments, with resident physicians achieving better results than specialists. The decisive role of a wide-ranging emergency medical basic training and the rapid accumulation of experience in younger prehospital emergency physicians with higher frequency of service for the correctness of the presumed diagnosis by prehospital emergency physicians was already noticed in other studies [15, 16]. This may also be suggested as an explanation for the better results for the correct drug administration by prehospital emergency resident physicians. The specialty and the approval for emergency medicine of prehospital emergency physicians did not influence the suspected diagnosis.

The proportion of 31% of missing DA varied in comparable studies on the correct diagnosis in EMS and in the emergency department with 9.9–35.9% [15–21]. Comparing the results, it has to be taken into account that the methods for determining the DA were different in these studies. There was a significant correlation between the incorrect suspected diagnosis and the medication error. Medication errors occurred in 21% of cases with a correct prehospital diagnosis and in 54% of cases with an incorrect prehospital diagnosis. It can be concluded that, in many cases, incorrect drug administration was a consequence of the incorrect suspected diagnosis.

A generalization of the results is limited due to the inhomogeneous emergency medical structure at the EMS centers over the German federal states. The informative value of the specialization for anaesthesiologists and general practitioners is limited due to their low proportion of the total number of prehospital emergency physician deployments.

## 5. Conclusion

The MA in prehospital emergency physician deployments according to the hospital discharge diagnosis as a quality feature in prehospital medical care shows a necessity for improvement with 31% medication errors. Incorrect diagnoses by the prehospital emergency physician seem to lead to medication errors in prehospital emergency physician deployments. The necessary standards and guidelines for administration of drugs should be taken into account in educational courses for prehospital emergency physicians. The wide-ranging emergency medical training and the rapid accumulation of operational experience seem to play a crucial role for correct administration of medication in the prehospital emergency physician deployments.

## Figures and Tables

**Table 1:** Listing of the deployment distribution according to specialty, medical educational status, and approval for emergency medicine of the prehospital emergency physician.

*Specialty*	
Internal medicine	453
Surgery	195
Anaesthesiology	9
General medicine	51

*Medical educational status*	
Specialist	227
Resident physician	481

*Approval for emergency medicine*	
Lower approval (“Fachkunde Rettungsdienst”)	467
Higher approval (“Zusatzbezeichnung Notfallmedizin”)	241

**Table 2:** The 10 most common administered drugs (*n* = 224) in prehospital emergency physician deployment with missing MA.

Drugs	Proportion, % (*n*)
Ringer solution	27% (59)
Heparin	13% (28)
Nitrospray	10% (21)
Furosemide	6% (14)
Morphine	6% (14)
Urapidil	5% (12)
Metoclopramid	4% (9)
Aspirin	4% (9)
Novalgin	4% (9)
Bayotensin	3.5% (8)

**Table 3:** Administered drugs with missing MA (*n* = 224), summarized in medication groups by ATC codes.

Medication groups (grouped by ATC codes), number (%)										
A	J	L	B	C	G	M	N	R	H	V
71 (32%)	0 (0%)	0 (0%)	37 (16%)	61 (27%)	0 (0%)	0 (0%)	34 (15%)	6 (3%)	4 (2%)	11 (5%)

*ATC codes*: A, alimentary tract and metabolism; J, anti-infectives for systemic use; L, antineoplastic and immunomodulating agents; B, blood and blood forming organs; C, cardiovascular system; G, genitourinary system and sex hormones; M, musculoskeletal system; N, nervous system; R, respiratory system; H, systemic hormonal preparations; V, various. ATC, anatomic therapeutic chemical classification.

**Table 4:** 10 most common discharge diagnoses (*n* = 708).

I10.91	Hypertensive crisis	9% (64)
I50.9	Cardiac decompensation	6% (40)
I63.9	Stroke	5% (35)
I21.4/I21.3/I21.9	Myocardial infarction	4.5% (33)
E16.2/E15	Hypoglycaemia/hypoglycaemic coma	4% (26)
G40.9	Seizure	3% (24)
E86	Exsiccosis	3% (20)
J15.9	Pneumonia	2.5% (19)
J44.09	Exacerbated COPD	2.5% (18)
I48.9	Arrhythmia absoluta	2.5% (17)

## Data Availability

The data used to support the findings of this study are available from the corresponding author upon reasonable request.

## Abbreviations

DIVI: Deutsche interdisziplinäre Vereinigung für Intensiv- und Notfallmedizin
DA: Diagnostic agreement
ED: Emergency department
IBM SPSS: International business machines corporation statistical package for the social science
ICD: International statistical classification of diseases and related health problems
MA: Medication appropriateness
SAP: Systeme, Anwendungen und Produkte in der Datenverarbeitung.

## References

[1] Ammenwerth E., Aly A. F., Bürkle T. (2014). Zum Einsatz von Informationstechnologie zur Verbesserung der Arzneimitteltherapiesicherheit (memorandum AMTS-IT). *GMS Medizinische Informatik, Biometrie und Epidemiologie*.

[2] Grandt D., Braun C., Häuser W. (2005). Häufigkeit, Relevanz, Ursachen und Strategien zur Vermeidung von Medikationsfehlern. *Zeitschrift für Gerontologie und Geriatrie*.

[3] Cox P. M., D’Amato S., Tillotson D. J. (2001). Reducing medication errors. *American Journal on Medical Quality*.

[4] Tully A. P., Hammond D. A., Li C., Jarrell A. S., Kruer R. M. (2019). Evaluation of medication errors at the transition of care from an ICU to non-ICU location. *Critical Care Medicine*.

[5] Lewis P. J., Dornan T., Taylor D., Tully M. P., Wass V., Ashcroft D. M. (2009). Prevalence, incidence and nature of prescribing errors in hospital inpatients: a systematic review. *Drug Safety*.

[6] Mende A. (2014). BfArM erfasst Medikationsfehler. *Pharmazeutische Zeitung*.

[7] González G. G., Morales L. M., de Miguel García S., Domínguez C. J., Pérez N. D., Herrera I. M. (2019). Descriptive analysis of medication errors notified by primary health care: learning from errors. *Atención Primaria*.

[8] Lesar T. S., Briceland L., Stein D. S. (1997). Factors related to errors in medication prescribing. *JAMA: The Journal of the American Medical Association*.

[9] Zarif-Yeganeh M., Rastegarpanah M., Garmaroudi G., Hadjibabaie M., Sheikh Motahar Vahedi H. (2017). Incidence of medication discrepancies and its predicting factors in emergency department. *Iranian Journal of Public Health*.

[10] Salameh L., Farha R. A., Basheti I. (2018). Identification of medication discrepancies during hospital admission in Jordan: prevalence and risk factors. *Saudi Pharmaceutical Journal*.

[11] Farha R. A., Hammour K. A., Al-Jamei S., AlQuda R., Zawiah M. (2018). The prevalence and clinical seriousness of medication discrepancies identified upon hospital admission of pediatric patients. *BMC Health Services Research*.

[12] van der Luit C. D., de Jong I. R., Ebbens M. M. (2018). Frequency of occurrence of medication discrepancies and associated risk factors in cases of acute hospital admission. *Pharmacy Practice*.

[13] Vilke G. M., Tornabene S. V., Stepanski B. (2007). Paramedic self-reported medication errors. *Prehospital Emergency Care*.

[14] Mogaka B., Clary D., Hong C., Farris C., Perez S. (2018). Medication reconciliation in the emergency department performed by pharmacists. *Baylor University Medical Center Proceedings*.

[15] Ramadanov N., Schlattmann P., Behringer W. (2018). Übereinstimmung zwischen notärztlicher Verdachtsdiagnose und Entlassungsdiagnose. *Notfall + Rettungsmedizin*.

[16] Peter J. Qualität notärztlicher Diagnosen: ein Vergleich von Fachärzten und Weiterbildungsassistenten der Anästhesie Bd. 4. https://opus4.kobv.de/opus4-fau/files/1170/Peter_Jochen_Dissertation.pdf.

[17] Ramadanov N., Klein R., Ramadanova N., Behringer W. (2019). Influence of time of mission on correct diagnosis by the prehospital emergency physician: a retrospective study. *Emergency Medicine International*.

[18] Ramadanov N., Klein R., Laue F., Behringer W. (2019). Diagnostic agreement between prehospital emergency and in-hospital physicians. *Emergency Medicine International*.

[19] Arntz H. R., Klatt S., Stern R., Willich S. N., Benecker J. (1997). Sind Notarztdiagnosen zuverlässig?. *Notfall + Rettungsmed*.

[20] Heuer J. F., Gruschka D., Crozier T. A. (2012). Accuracy of prehospital diagnoses by emergency physicians: comparison with discharge diagnosis. *European Journal of Emergency Medicine*.

[21] Dormann H., Diesch K., Ganslandt T., Hahn E. G. (2010). Kennzahlen und qualitätsindikatoren einer medizinischen notaufnahme. *Deutsches Aerzteblatt Online*.

